# Utilisation of health services and the poor: deconstructing wealth-based differences in facility-based delivery in the Philippines

**DOI:** 10.1186/s12889-016-3148-0

**Published:** 2016-07-06

**Authors:** Andrew Hodge, Sonja Firth, Raoul Bermejo, Willibald Zeck, Eliana Jimenez-Soto

**Affiliations:** School of Public Health, The University of Queensland, Public Health Building, Herston Road, Herston, Brisbane, Queensland 4006 Australia; UNICEF Philippines Country Office, Manila, Philippines; Institute of Tropical Medicine, Antwerp, Belgium; Department of Obstetrics and Gynaecology, Medical University of Graz, Graz, Austria

**Keywords:** Facility-based delivery care, Wealth-based coverage gaps, Decomposition, The Philippines

## Abstract

**Background:**

Despite achieving some success, wealth-related disparities in the utilisation of maternal and child health services persist in the Philippines. The aim of this study is to decompose the principal factors driving the wealth-based utilisation gap.

**Methods:**

Using national representative data from the 2013 Philippines Demographic and Health Survey, we examine the extent overall differences in the utilisation of maternal health services can be explained by observable factors. We apply nonlinear Blinder-Oaxaca-type decomposition methods to quantify the effect of differences in measurable characteristics on the wealth-based coverage gap in facility-based delivery.

**Results:**

The mean coverage of facility-based deliveries was respectively 41.1 % and 74.6 % for poor and non-poor households. Between 67 and 69 % of the wealth-based coverage gap was explained by differences in observed characteristics. After controlling for factors characterising the socioeconomic status of the household (i.e. the mothers’ and her partners’ education and occupation), the birth order of the child was the major factor contributing to the disparity. Mothers’ religion and the subjective distance to the health facility were also noteworthy.

**Conclusions:**

This study has found moderate wealth-based disparities in the utilisation of institutional delivery in the Philippines. The results confirm the importance of recent efforts made by the Philippine government to implement equitable, pro-poor focused health programs in the most deprived geographic areas of the country. The importance of addressing the social determinants of health, particularly education, as well as developing and implementing effective strategies to encourage institutional delivery for higher order births, should be prioritised.

## Background

Many low- and middle-income countries are beset by wealth-based disparities in the use of reproductive, maternal, newborn and child health (RMNCH) services and outcomes [1–3]. Evidence of persistent socioeconomic inequalities has spurred attempts by governments and international donors to address the delivery of health services to the poor and disadvantaged [4–7]. An established stylised fact is that mothers from higher socioeconomic backgrounds are more likely to deliver in health facilities [1, 3]. Yet, very little is known about the factors that contribute to these inequalities. Such factors could clearly inform targeted government interventions.

In the Philippines, substantial variations in health services and outcomes continue across wealth quintiles despite national reductions in child mortality rates [2, 3]. The country faces significant challenges arising from its geography, income distribution and susceptibility to natural disasters [8]. While levels of coverage for many RMNCH services have increased across the nation, the quality of care is inadequate, particularly for lower socioeconomic women [2, 9]. Recent data, for example, suggest that the percentage of women from the richest quintile delivering their child with a skilled provider was 96 %, compared to only 42 % for women in the poorest quintile [9]. Such discrepancies are common in the Philippines and the factors responsible for the wealth-based utilisation gaps remain unknown. In this study, we focus on the utilisation of institutional birth delivery. This service is a complex intervention provided for a standard purpose and recommended for all women. It requires skilled labour and considerable capital in order to provide 24-h functioning facilities. Consequently, institutional delivery can usefully represent facility-based care for RMNCH more generally.

The aim of the paper is to examine the extent to which observable factors explain the overall differences in the utilisation of facility-based delivery. To address this question we used data from the 2013 Philippines Demographic and Health Survey (DHS). The survey captures a wide range of variables on the utilisation of health services and collects basic demographic, socioeconomic and health data. A recent study [3] similarly sought to assess the contributions made by household and individual factors to wealth-related disparities in the use of institutional delivery services. However, that study utilised the 2008 wave of the DHS and decomposed concentration indices. We apply a Blinder-Oaxaca decomposition analysis [10, 11]. Initially developed in the labour-economics literature, the Blinder-Oaxaca technique has gained some popularity in research related to health services and outcomes [12–15]. The method is employed to decompose wealth-based differences in facility-based delivery into the portion attributable to differences in observable characteristics and the part due to other factors. To account for the binary dependent variable, we utilise a nonlinear variation on the decomposition method introduced by Fairlie [16, 17].

## Methods

### Data

Data from the 2013 Philippines DHS were utilised for the purpose of this study [9]. The tenth in a series of cross-section surveys conducted by the Philippine National Statistics Office, it utilised a stratified two-stage cluster sampling scheme and provided representative population descriptions at the national and provincial levels as well as for urban and rural areas. The survey sampled women aged 15–49 years, with a barangay (i.e. the smallest administrative unit in the country equivalent to a village, district or ward) or part of a barangay selected as primary sampling units. Fieldwork was conducted from August to September 2013. A total of 14,804 households were surveyed at a response rate of 99.4 %. Among the households interviewed a total of 16,155 women were surveyed, of whom 10,125 have had children. A number of questions pertaining to pre-natal and post-natal health care and services are limited to the mother’s last birth to have occurred in the five years preceding the survey. This yields a total sample of 7,216 birth-observations, with missing data reducing the sample to 7,121; with 2,266, 1,607, 1,334, 1,099 and 815 observations from the lowest to richest wealth quintiles, respectively. Full details on the survey design and implementation are available elsewhere [9]. The publicly available dataset was obtained through online resources. The data were anonymous, with no identifiable information on the survey participants. As such internal review approval was not required.

### Variables

In the analysis the outcome variable is facility-based delivery. It was constructed drawing on the survey question: “Where did you give birth to [name of child]?” Mothers are given the option of selecting from a list of places under the headings of home, public sector, private sector or other. We define a dichotomous variable whereby a birth is categorised as facility-based if it was known to have occurred at a private, public or non-governmental clinic. The focus on facility-based delivery was motivated by several considerations. First, this variable is an objective rather than subjective measure given that all women having a delivery are in need of the service. Second, facility-based delivery is the best proxy measure of skilled birth attendance, particularly given potential recall bias. This health service is essential and is included in the universal coverage agenda and a priority for coverage scale-up given its high efficacy for the prevention of maternal and neonatal mortality [18, 19]. Third, facility-based delivery is an intervention capable of representing both the ability of the health system to supply a complex, skilled service and the women’s ability to utilise services even under the difficult circumstance of childbirth. We also note that the sociocultural and traditional factors influencing health care have been known to differ between men and women in other contexts and women are generally the primary actors in care-seeking for ill children [4, 20–25]. Consequently, drawing on women’s responses rather than on the men’s questionnaire is more reliable in capturing the barriers to the utilisation of RMNCH facility-based services.

The decomposition is based on a split of the sample into poor and non-poor groups based on an asset-based wealth index constructed using principal component analysis [26]. This survey-provided index is used to group sampled households into thirds. Official national estimates put poverty at approximately 25 % of the population in the Philippines [27]. Moreover, a number of poverty alleviation programs of the government – including Conditional Cash Transfers (CCT), housing and enrolment into the national health insurance program (PhilHealth) – target the bottom two income quintiles of the population. Consequently, based on the wealth index, we define the poor and non-poor groups as the bottom two quintiles and as the top three quintiles of the sample, respectively.

The choice of covariates was motivated by three factors. First, we sought to test the significance of multiple factors. Second, it was guided by previous empirical studies, which have focused on accessibility and the sociocultural factors associated with facility-based delivery [28–31]. Third, the choice was limited by the availability of relevant variables. Consequently, various maternal, socioeconomic and sociocultural factors were included in the model.

To isolate the influence of other variables it is important to first control for various socioeconomic factors. We used several variables. Mothers’ and her partner’s education were used as proxies for knowledge and awareness of health issues. At least weekly viewing of television captured access to information. Formal employment might similarly increase the range and access of information available to mothers. Furthermore, employment might provide mothers with the means to have a facility delivery. On the other hand, the opposite effect might occur if employment is poverty-induced and indicate resource constraints. Both the mother’s and her partner’s employment are included. Despite being limited by the available data, we maintain that these variables are likely to be highly associated with wealth and are important base control variables.

Geographic accessibility was captured through one subjective binary (0/1) indicator of the distance to a health facility according to respondent’s answer to the question “when sick and wanting to get medical advice or treatment the distance to the health facility is a big problem”. Various maternal characteristics were included as control variables in order to separate the influence from other covariates, especially employment and education. The included characteristics were the mother’s fertility levels represented by birth order of the child, whether the child is a twin/triplet and the mother’s pregnancy history (i.e. pregnancy termination and age at birth). Furthermore, two sociocultural factors were included: marital status and religion. Each may influence the choice of delivery place via the influence of female autonomy, social norms, beliefs and values, and possible discrimination [28]. Table 1 and Appendix Table 5 provide definitions and descriptive statistics of all explanatory variables used in the analyses.

**Table 1:** Descriptive statistics by wealth group

Variable	Poor (P)		Non-Poor (NP)				
	Mean	S.D.	Mean	S.D.	Differential NP-P	S.E.	
*Dependent variables*							
Facility-based delivery	0.41	(0.49)	0.75	(0.44)	0.34	(0.02)	***
*Supply factor*							
Distance to health facility	0.43	(0.49)	0.20	(0.40)	−0.23	(0.02)	***
*Socioeconomic factors*							
Mother’s Education:							
None	0.03	(0.17)	0.002	(0.04)	−0.03	(0.005)	***
Incomplete Primary	0.20	(0.40)	0.021	(0.14)	−0.18	(0.01)	***
Complete Primary	0.16	(0.37)	0.052	(0.22)	−0.11	(0.01)	***
Secondary or more	0.61	(0.49)	0.925	(0.26)	0.32	(0.02)	***
Partner’s Education:							
None	0.04	(0.19)	0.003	(0.06)	−0.03	(0.006)	***
Incomplete Primary	0.29	(0.45)	0.03	(0.18)	−0.25	(0.01)	***
Complete Primary	0.18	(0.38)	0.06	(0.23)	−0.12	(0.01)	***
Secondary or more	0.48	(0.50)	0.86	(0.35)	0.38	(0.01)	***
Mother watches television	0.57	(0.49)	0.91	(0.29)	0.33	(0.02)	***
Mother’s Employment							
Agriculture	0.16	(0.36)	0.02	(0.13)	−0.14	(0.01)	***
None	0.56	(0.50)	0.46	(0.50)	−0.10	(0.02)	***
Manual	0.09	(0.28)	0.10	(0.31)	0.02	(0.01)	
Professional	0.19	(0.40)	0.42	(0.49)	0.23	(0.01)	***
Partner’s Employment							
Agriculture	0.49	(0.50)	0.09	(0.29)	−0.40	(0.02)	***
None	0.01	(0.11)	0.02	(0.13)	0.01	(0.004)	
Manual	0.38	(0.49)	0.46	(0.50)	0.08	(0.02)	***
Professional	0.10	(0.30)	0.39	(0.49)	0.28	(0.01)	***
*Maternal factors*							
Multiple birth	0.01	(0.12)	0.01	(0.12)	−0.00	(0.004)	
Previously term. preg.	0.18	(0.39)	0.15	(0.36)	−0.03	(0.01)	**
Child’s birth order							
1	0.22	(0.42)	0.39	(0.49)	0.17	(0.01)	***
2-4	0.50	(0.50)	0.53	(0.50)	0.03	(0.01)	**
> 4	0.28	(0.45)	0.09	(0.28)	−0.19	(0.01)	***
Mother’s age at birth							
15-19	0.12	(0.33)	0.12	(0.33)	0.00	(0.01)	
20-29	0.50	(0.50)	0.52	(0.50)	0.02	(0.01)	
30-49	0.38	(0.48)	0.36	(0.48)	−0.02	(0.01)	
*Sociocultural factors*							
Religion							
Catholic	0.71	(0.45)	0.84	(0.36)	0.13	(0.02)	***
Protestant	0.06	(0.25)	0.04	(0.19)	−0.03	(0.01)	***
Islam	0.12	(0.33)	0.03	(0.16)	−0.09	(0.02)	***
Other	0.11	(0.31)	0.09	(0.29)	−0.01	(0.01)	
Mother’s Marital Status							
Married	0.67	(0.47)	0.59	(0.49)	−0.08	(0.02)	***
Living together	0.29	(0.46)	0.30	(0.46)	0.01	(0.02)	
Other	0.04	(0.20)	0.11	(0.31)	0.07	(0.01)	***
*Geography*							
NCR	0.04	(0.19)	0.25	(0.44)	0.22	(0.02)	***
CAR	0.01	(0.10)	0.02	(0.14)	0.01	(0.004)	**
Ilocos	0.05	(0.21)	0.04	(0.21)	−0.00	(0.01)	
Cagayan Valley	0.04	(0.20)	0.03	(0.18)	−0.01	(0.01)	
Central Luzon	0.06	(0.23)	0.13	(0.33)	0.07	(0.01)	***
CALABARZON	0.07	(0.25)	0.18	(0.39)	0.12	(0.02)	***
MIMAROPA	0.04	(0.20)	0.01	(0.12)	−0.03	(0.01)	***
Bicol	0.09	(0.28)	0.04	(0.20)	−0.04	(0.01)	***
Western Visayas	0.09	(0.29)	0.05	(0.21)	−0.05	(0.01)	***
Central Visayas	0.08	(0.27)	0.05	(0.23)	−0.02	(0.01)	*
Eastern Visayas	0.05	(0.22)	0.03	(0.16)	−0.03	(0.01)	**
Zamboanga Peninsula	0.07	(0.25)	0.03	(0.17)	−0.04	(0.01)	***
Northern Mindanao	0.07	(0.25)	0.03	(0.16)	−0.04	(0.01)	***
Davao Peninsula	0.07	(0.25)	0.05	(0.21)	−0.02	(0.01)	*
SOCCSKSARGEN	0.07	(0.25)	0.03	(0.16)	−0.04	(0.01)	***
Caraga	0.04	(0.21)	0.02	(0.15)	−0.02	(0.01)	***
ARMM	0.07	(0.26)	0.004	(0.06)	−0.07	(0.01)	***
Obs.	3,873		3,248				

*Notes:* Poor and non-poor groups are defined by the bottom two quintiles and top three quintiles, respectively. Differentials are proportional differences. Tests on the equality of proportions are two-tailed and (*), (**) and (***) represent statistical significance at 10 %, 5 % and 1 % levels, respectively. S.D., standard deviation; S.E., standard error; Obs., observations; term. preg., terminated pregnancy

### Statistical analysis

Wealth-based differences in the coverage of facility-based delivery care were assessed using a regression-based decomposition approach. In principle, the decomposition isolates the share of the coverage gap in the outcome variable due to differences in observable factors across the groups, which in our case are defined by the wealth index. While first developed for the unbounded continuous dependent variable case by Oaxaca [10] and Blinder [11], we used a binary dependent variable extension of the Blinder-Oaxaca method formulated by Fairlie [16, 17].

The decomposition involves two main steps. First, an appropriate probability model to link the outcome variable to the set of independent variables for each group is estimated. We opt to use wealth-specific logit models of the form:

1 $$ {Y}_i^{*}={\mathbf{X}}_i^{\mathbf{\prime}}{\beta}^J+{\varepsilon}_i;\kern2em {Y}_i=1\kern0.5em \mathrm{if}\kern0.5em {Y}_i^{*}>0\kern1em \mathrm{and}\kern1em {Y}_i=0\kern0.5em \mathrm{if}\kern0.5em {Y}_i^{*}\le 0 $$

where *Y*_*i*_ is facility-based delivery – which takes a value of 1 if birth *i* took place in a facility, 0 otherwise. The vector **X**_*i*_ contains the independent variables and *ε*_*i*_ is the error term, which is assumed to be logistic, independent of the covariates, and independent for children in different communities that constitute the survey’s primary sample unit. The parameter vectors *β*^*J*^ are to be estimated separately for each sub-sample (i.e. *J* = *P* for the poor and *J* = *NP* for the non-poor) and using the pooled sample.

Second, the average regional difference in *Y* is decomposed as:

2 $$ \begin{array}{cc}\hfill {\overline{Y}}^{NP}-{\overline{Y}}^P=\hfill & \hfill \left[{\displaystyle \sum_{i=1}^{N^{NP}}\frac{F\left({\mathbf{X}}_i^{NP}{\widehat{\beta}}^{NP}\right)}{N^{NP}}-{\displaystyle \sum_{i=1}^{N^P}\frac{F\left({\mathbf{X}}_i^P{\widehat{\beta}}^{NP}\right)}{N^P}}}\right]\hfill \\ {}\hfill \hfill & \hfill +\left[{\displaystyle \sum_{i=1}^{N^P}\frac{F\left({\mathbf{X}}_i^P{\widehat{\beta}}^{NP}\right)}{N^P}-{\displaystyle \sum_{i=1}^{N^P}\frac{F\left({\mathbf{X}}_i^P{\widehat{\beta}}^P\right)}{N^P}}}\right]\hfill \end{array} $$

where *N*^*J*^ is the sample size, ${\overline{Y}}^J$ is the average probability of the outcome variable, **X**_*i*_^*J*^ is a row vector of independent variables of observation *i* and *β*^*J*^ is a vector of logit coefficient estimates (including the intercept), all for the *J* wealth-based households. The first term in equation (2) measures the proportion of the wealth gap that is due to group differences in the distributions of **X** (i.e. the “explained” portion or the endowment effect). The second term signifies the part due to differences in the group processes determining the levels of *Y* and the group differences in unmeasurable or unobserved endowments (i.e. the “unexplained” portion). The latter term is often interpreted as reflecting unobservable factors, which can include group-specific attitudes or omitted variables [14].

It is worth noting four methodological points related to the decomposition. First, it is equally valid to formulate the decomposition by replacing ${\widehat{\beta}}^{NP}$ in the first term with ${\widehat{\beta}}^P$ and substitute *N*^*P*^ and **X**_*i*_^*P*^ with *N*^*NP*^ and **X**_*i*_^*NP*^ in the second term. According to this specification we would be using the parameters from the poor sub-sample as the weights or “benchmark” in the first term of the decomposition. The benchmarking will provide a completely different set of estimates. This is a well-known problem with Blinder-Oaxaca decomposition. Moreover, alternatively we could weigh the first term of the decomposition using the coefficient estimates from the pooled sample of the two groups. Thus, to test the sensitivity to the choice of coefficients, we present estimates using the two groups as benchmarks along with the pooled parameters (presented in the Appendix).

Second, it is possible to further decompose the explained portion into the contributions of each covariate or groups of covariates in the case of categorical variables (e.g. mother’s education). This detailed decomposition requires one-to-one matching of observations from both wealth-based groups. However, the results are potentially sensitive to the matching procedure. To address this, we randomly draw 100 samples from the larger non-poor sub-sample to match the poor sample, and the results are reported as means across the simulations. Third, contribution of each covariate is conditional on the contribution of the previous covariate [17]. Consequently, the results are influenced by the ordering of the covariates in the specification. Accordingly, in each replication we randomise the ordering of the independent variables to minimise the impact of this arbitrariness. Finally, it should be noted that in the presence of categorical variables, the choice of the omitted reference category does not influence the detailed decomposition [32]. Using the provided sample weights, the computations and the matching procedure account for the survey structure. All statistical analyses are estimated in Stata® 13.

## Results

### Descriptive results

Table 1 presents the mean values of the variables used in the decomposition analysis. The descriptive statistics show higher mean coverage of facility-based delivery amongst the rich compared to the poor; the raw differential is 33.6 percentage-points, as shown in the top panel of Table 1. The difference is statistically significant at conventional levels.

The descriptive statistics by wealth groups also shed light on the differences in the observable characteristics. The distance to a health facility is reported to be a larger problem amongst the poor. Unsurprisingly socioeconomic status favours the rich. Comparing the percentages in the higher categories, mothers from richer households tend to be more highly educated and employed in professional/service industries: approximately 61 % of poor mothers have attained secondary or higher level education compared to approximately 93 % of mothers from rich households. Similarly, partners from poorer households tend to work in agriculture and have mostly primary education levels. Mothers from poorer households tend to report less access to information: 57 % of poor mothers report watching television at least once a week compared to 91 % for richer households. A higher percentage of poorer households are non-Catholic, while a substantial percentage of richer households live in the National Capital Region. Overall, the descriptive statistics suggest noticeable wealth-based differences in the outcome variable and the characteristics of the women and households.

### Decomposition results

The results of the decomposition of the observed differences in the coverage of facility-based delivery between poor and non-poor households are reported in Table 2. We also estimated the model using a linear probability model and the results are similar. Since the results may be sensitive to the choice of benchmark parameters (i.e. counterfactuals), we present both the results using either wealth groups’ coefficients as the weights. The Appendix includes the decomposition results based on the pooled sample coefficients and the baseline logit estimates. Across the wealth groups, the odds-ratios and tests of statistical significance tend to be similar. A notable exception is the reduced odds and statistical significance for education from non-poor households.

**Table 2:** Non-linear decomposition of the difference in facility-based delivery between wealth groups

${\overline{Y}}_{NP}$	0.746							
${\overline{Y}}_P$	0.411							
Wealth gap $\left({\overline{Y}}_{NP}-{\overline{Y}}_P\right)$	0.336							
Benchmark Coefficients	Non-Poor				Poor			
	Decomp.		S.E.	Cont. (%)	Decomp.		S.E.	Cont. (%)
*Supply-side*								
Distance to facility	0.014	**	(0.006)	4.06	0.011	***	(0.004)	3.42
*Socioeconomic factors*								
Mother’s education	0.017		(0.015)	4.93	0.026	***	(0.007)	7.79
Partner’s education	0.025	*	(0.015)	7.49	0.031	***	(0.009)	9.14
Mother watches TV	0.011		(0.011)	3.20	0.028	***	(0.006)	8.48
Mother’s employment	0.028	***	(0.009)	8.22	0.020	***	(0.006)	5.87
Partner’s employment	0.065	***	(0.013)	19.29	0.037	***	(0.010)	11.09
*Maternal factors*								
Multiple birth	−0.0005		(0.0005)	−0.14	−0.001		(0.000)	−0.16
Previously term. preg.	0.0003		(0.001)	0.10	0.001		(0.001)	0.30
Child’s birth order	0.058	***	(0.008)	17.26	0.042	***	(0.006)	12.59
Mother’s age at birth	−0.003		(0.001)	−0.82	−0.001	*	(0.001)	−0.38
*Sociocultural factors*								
Religion	0.007		(0.005)	2.19	0.014	***	(0.004)	4.18
Marital status	−0.007	**	(0.003)	−2.00	0.003		(0.004)	0.79
*Geography*								
Regions	0.020	**	(0.010)	6.01	0.013		(0.017)	3.95
Total explained	0.234			69.78	0.225			67.12
Total unexplained	0.101			30.22	0.110			32.88

*Notes:* Total number of observations is 7,121. Specifications include a dummy for missing partner information. The contribution of each variable to the regional gap is given under the column “Decomp.” computed as the mean values of the decomposition using 100 random non-poor income sub-samples with the order of the variables in each replication randomised. Standard errors are given in parentheses and (*), (**) and (***) represent statistical significance at 10 %, 5 % and 1 % levels, respectively. Decomp., decomposition; S.E., standard error; Cont., contribution; term. preg., terminated pregnancy

The overall wealth-based difference in the coverage of facility-based deliveries is a sizeable 33.6 percentage points, as shown in the top panel of Table 2. Of those percentage points, between 22.5 and 23.4 percentage points can be explained by the measureable characteristics specified in the model, depending on the chosen benchmark parameters. Accordingly, approximately 67–69 % of the wealth-based gap in coverage can be explained by the differences in average observed characteristics between poor and non-poor households. While we have been relatively successful in capturing the main factors explaining the disparity, at least 30 % of the gap remains unidentified.

The extent that the differences in specific observable characteristics are associated with the coverage gap is estimated via the detailed decomposition. All three socioeconomic factors are found to make statistically significant contributions. The proportion of the coverage gap accounted for by mothers’ (between 5 and 7.8 %) and their partners’ education (between 7.5 and 9.1 %), weekly television viewership (between 3.2 and 8.5 %), and mothers’ (between 5.9 and 8.2 %) and partners’ employment (between 11.1 and 19.3 %) are substantial. While statistically significant, the socioeconomic status is not the sole factor in explaining the coverage gap. Distance to a health facility is found to explain 1.1–1.4 percentage points of the 33.6 percentage point regional gap, approximately 3.4–4.1 % of the wealth-based difference in facility-based deliveries depending on the parameters used as the benchmarks. Regional differences (between 4 and 6 %) also make a sizable contribution.

One of the largest contributors to the wealth-based gap was the birth order of the child. This factor explained between 12 and 17 % of the gap. This contribution is a combination of the higher odds of having a facility-based delivery if the child is the first born and the higher percentage of first born children amongst the non-poor group. All other maternal factors made negligible contributions. The decomposition using the pooled samples coefficients provides a sensitivity check for these results. The proportions were found to be consistent and contributions similar in terms of statistical significance (see Appendix Table 5). Hence, while some results are sensitive to the choice of benchmark the overall conclusions are robust.

## Discussion

In this study we utilised recently released micro-level nationally representative data from the Philippines to examine the factors that explain the wealth-based disparities in the coverage of delivery care health services. We examined a range of socioeconomic, maternal and sociocultural factors as well as geography and the difficulty of accessing services to decompose the disparities. The results show that differences in observed characteristics explain 67–69 % of wealth-based gap, depending on the parameters used to weight the distribution of the characteristics. The largest contributors were the education and employment status of the parents and the birth order of the child. These results suggest that the perceived benefit of facility care and the financial ability of households to access such care appear to drive the wealth-based disparity in levels of facility-based deliveries.

As to be expected, inequalities in the socioeconomic status of the households represented the largest set of contributors to the wealth-based difference in facility-based delivery. More educated partners are likely to have more open attitudes towards modern medicine, more awareness of the benefits of facility-based care and more capability of demanding the type of care they deem adequate [28, 33]. Higher levels of education are likely to be associated with access to the financial resources (either directly or through knowledge of government programs and insurance schemes) to seek institutional care. On the other hand, the reduced odds and significance of education for non-poor households is unsurprising given the lack of variation in education across sampled non-poor households, with the vast majority having obtained secondary or higher education.

Economic accessibility likely underlies the contribution of the partners’ occupation. Households less reliant on agriculture are found to have higher probability to seek a facility-based delivery. This likely reflects the higher costs of facility delivery for those engaged in agriculture [34]. Such costs will include not only transportation costs but income forgone [35].

Even after controlling for socioeconomic status, the birth order of the child remained a large contributor to the gap. The percentage of first time mothers was higher amongst richer households and such mothers are more likely to have a facility-based delivery. This may reflect the uncertainty associated with the first pregnancy, with women with no previous experience of delivery more likely to be encouraged by health workers to have an institutional delivery [36]. More experienced mothers with histories of uncomplicated deliveries may believe professional care is unnecessary, particularly if it involves substantial costs in the form of child care and forgone household income [37]. The disparity due to birth order may also be an effect of insurance benefit coverage policies of Philhealth. The national insurer progressively covered up to the second delivery in 2003 [38], the third delivery in 2006 [39], the 4th delivery in 2008 [40], and all deliveries regardless of birth order in 2014 [41].

Subjective distance to a health facility and religion were found to be small but significant contributors to the disparities. Distance can act as disincentive to seeking care, particularly if there is a lack of transportation or the quality of care is perceived to be poor [28, 42, 43]. Yet, its impact is not substantial. This suggests that factors, such as affordability, acceptability, awareness, knowledge and attitudes, work in tandem with geographic accessibility as barriers to utilisation [44]. The contribution of religion is driven by statistically significant lower odds of Muslim mothers seeking delivery care and higher percentage of poor Muslims households. It is difficult to exactly isolate what drives this contribution but it is important to further examine the role of conflict and security. Most Muslim Filipinos reside in areas with on-going insurgencies. In the Muslim-dominated and conflict-ridden ARMM region, the proportion of women that delivered in a health facility is the lowest in the country, at 12.3%. Conflict has complex social effects that may impact on access to facility-based delivery care services [45, 46]. It is also possible that such groups are discriminated against by staff as is the case in other contexts [47], poor health infrastructure and transport may exist in areas where minorities live [48] or some cultural requirement might make mothers avoid an institutional delivery [28].

Several caveats relate to the findings of this study. First, it should be noted that causal relationships cannot be asserted without longitudinal datasets and natural experiments. Extensions of this work should attempt to estimate a causal model to verify these results. Second, geographic accessibility was captured only through one subjective indicator of the distance to a health facility. Principally, this variable relies on respondents’ *perceived* need for health services. Moreover, women are only asked to assess the difficulty in reaching a health facility. This does not necessarily represent the difficulty in reaching either a facility that can do a delivery or a facility known to offer quality services. Nonetheless, it does more widely represent barriers to health service utilisation by women for any illness. Third, data limitations entailed the use of proxies for potentially important factors that contribute to the wealth-based gap. No direct measure of quality of care, for example, is available. Although we control for a great many correlates and have relied on previous studies to specify the model, possible omitted variable bias cannot be ruled out.

## Conclusion

This study has found moderate wealth-based disparities in the utilisation of institutional delivery in the Philippines. The results suggest that the largest contributors to this gap were the inequalities associated with the parents’ education and employment status and the birth order of the child. Our findings confirm the importance of recent efforts made by the Philippine government to implement equitable, pro-poor focused health programs in the most deprived geographic areas of the country. They also highlight the importance of addressing the social determinants of health such as education. Although recent gains in education might contribute to narrowing this gap, concerted effort to improve institutional deliveries for higher order births is needed. The recent increase in the number of the poor covered by national health insurance (from 5.2 million families at the time of the survey to 14.7 million families in 2014 [49]) will likely impact on the economic barriers but does not address issues of income security, particularly those in the agriculture sector. Finally, the importance of birth-order on wealth-related disparities for institutional delivery highlights the need for the country to effectively implement the Responsible Parenthood and Reproductive Health Law [50] to address significant unmet needs for family planning, particularly among poor women.

## Abbreviations

CCT, Conditional Cash Transfers; DHS, Demographic and Health Survey; RMNCH, Reproductive, Maternal, Newborn and Child Health

## Appendix

**Table 3:** Logit regressions for facility-based delivery

Specification	(1)		(2)		(3)	
Sample used	Non-Poor		Poor		Pooled	
*Supply-side*						
Distance to facility	0.739	***	0.780	***	0.757	***
	(0.09)		(0.07)		(0.06)	
*Socioeconomic factors*						
Mother’s education (omitted: none)						
Incomplete Primary	0.979		1.720		1.634	
	(1.22)		(0.77)		(0.65)	
Complete Primary	1.058		2.098		1.960	*
	(1.27)		(0.98)		(0.80)	
Secondary or more	1.312		2.690	**	2.493	**
	(1.53)		(1.24)		(1.00)	
Partner’s education (omitted: none)						
Incomplete Primary	0.532		1.478		1.247	
	(0.40)		(0.52)		(0.37)	
Complete Primary	0.503		1.394		1.179	
	(0.35)		(0.52)		(0.36)	
Secondary or more	0.694		1.997	*	1.675	*
	(0.48)		(0.71)		(0.49)	
Mother watches TV	1.184	**	1.519	***	1.397	***
	(0.20)		(0.14)		(0.11)	
Mother’s Employment (omitted: agriculture)						
None	2.327	***	1.694	***	1.739	***
	(0.72)		(0.25)		(0.24)	
Manual	2.444	***	1.632	**	1.750	***
	(0.82)		(0.34)		(0.30)	
Professional	2.496	***	1.884	***	1.931	***
	(0.77)		(0.31)		(0.27)	
Partner’s Employment (omitted: agriculture)						
None	3.269	**	1.895		2.400	***
	(1.51)		(0.77)		(0.68)	
Manual	1.615	***	1.315	**	1.387	***
	(0.27)		(0.14)		(0.13)	
Professional	2.496	***	1.705	***	2.166	***
	(0.51)		(0.27)		(0.25)	
*Maternal factors*						
Multiple birth	2.753	*	3.970	***	3.548	***
	(1.44)		(1.68)		(1.25)	
Previously terminated pregnancy	0.943		0.849		0.882	
	(0.13)		(0.10)		(0.08)	
Child’s birth order (omitted: 1)						
2-4	0.524	***	0.445	***	0.481	***
	(0.06)		(0.05)		(0.04)	
> 4	0.209	***	0.329	***	0.286	***
	(0.04)		(0.05)		(0.03)	
Mother’s age at birth (omitted: 15–19)						
20-29	1.035		1.381	**	1.218	*
	(0.17)		(0.20)		(0.14)	
30-49	1.749	***	1.948	***	1.888	***
	(0.34)		(0.35)		(0.25)	
*Sociocultural factors*						
Religion (omitted: Catholic)						
Protestant	1.401		0.781		0.969	
	(0.38)		(0.18)		(0.16)	
Islam	0.573		0.487	**	0.526	**
	(0.16)		(0.12)		(0.10)	
Other	1.139		0.740	**	0.899	
	(0.11)		(0.10)		(0.12)	
Marital status (omitted: married)						
Living together	0.822		0.913		0.861	*
	(0.11)		(0.10)		(0.07)	
Other	0.628	**	1.203		0.772	
	(0.13)		(0.31)		(0.12)	
Poorest					0.597	***
					(0.05)	
Pseudo *R* ^*2*^	0.110		0.146		0.198	
Obs.	3,248		3,873		7,121	

*Notes:* Estimated odd-ratios are reported with standard errors in parentheses. (*), (**) and (***) represent statistical significance at 10 %, 5 % and 1 % levels, respectively. All specifications include regional dummies and a dummy for missing partner information

**Table 4:** Non-linear decomposition of the difference in facility-based delivery between wealth groups, pooled sample benchmark

${\overline{Y}}_{NP}$	0.746			
${\overline{Y}}_P$	0.411			
Regional gap $\left({\overline{Y}}_{NP}-{\overline{Y}}_P\right)$	0.336			
Benchmark Coefficients	Pooled			
	Decomp.		S.E.	Cont. (%)
*Supply-side*				
Distance to facility	0.013	***	(0.004)	3.81
*Socioeconomic factors*				
Mother’s education	0.026	***	(0.007)	7.77
Partner’s education	0.032	***	(0.007)	9.46
Mother watches TV	0.023	***	(0.005)	6.71
Mother’s employment	0.021	***	(0.005)	6.27
Partner’s employment	0.049	***	(0.008)	14.72
*Maternal factors*				
Multiple birth	−0.0004		(0.0003)	−0.10
Previously term. preg.	0.0007		(0.0005)	0.21
Child’s birth order	0.044	***	(0.005)	13.05
Mother’s age at birth	−0.002		(0.001)	−0.62
*Sociocultural factors*				
Religion	0.012	***	(0.004)	3.58
Marital status	−0.004	*	(0.002)	−1.15
*Geography*				
Regions	0.019	**	(0.009)	5.55
Total explained	0.232			69.23
Total unexplained	0.103			30.77

*Notes:* Total number of observations is 7,121. Specifications include a dummy for missing partner information. Standard errors are given in parentheses and (*), (**) and (***) represent statistical significance at 10 %, 5 % and 1 % levels, respectively. Decomp., decomposition; S.E., standard error; Cont., contribution; term. preg., terminated pregnancy

**Table 5:** Variable descriptions

Variable	Definition
Facility-based delivery	(0/1) if the birth took place in a health facility (private, public or nongovernmental)
Wealth	Wealth quintiles derived using principal components analysis and household assets
Distance to health facility	(0/1) if the mother responded to the question “Many different factors can prevent women from getting medical advice or treatment for themselves. When you are sick and want to get medical advice or treatment, is the distance to the health facility” a big problem
Mother’s education:	Mother’s education attainment: (1) None; (2) Incomplete Primary; (3) Complete Primary; (4) Secondary or more
Partner’s education:	Partner’s education attainment: (1) None; (2) Incomplete Primary; (3) Complete Primary; (4) Secondary or more
Mother watches TV	(0/1) if the mother watches television at least once a week
Mother’s employment	Mother’s employment status: (1) Agriculture; (2) None; (3) Manual; (4) Professional/Services
Partner’s employment	Partner’s employment status: (1) Agriculture; (2) None; (3) Manual; (4) Professional/Services
Multiple birth	(0/1) if the child is a twin, triplet, etc.
Previously terminated pregnancy	(0/1) if the mother ever had a terminated pregnancy
Child’s birth order	Birth rank of the child: (1) first-born child of the woman; (2) 2nd to 4th child; (3) child of birth order 5 or more
Mother’s age at birth	Mother’s age at the birth of the child: (1) 15–19; (2) 20–29; (3) 30–49
Mother’s marital status	Mother marital status: (1) Married; (2) Living together; (3) Other
Religion	Mother’s religion: (1) Catholic; (2) Protestant; (3) Islam; (4) Other

## References

[1] Houweling TA, Ronsmans C, Campbell OM (2007). Huge poor-rich inequalities in maternity care: an international comparative study of maternity and child care in developing countries. Bull World Health Organ.

[2] Kraft AD, Nguyen KH, Jimenez-Soto E (2013). Stagnant neonatal mortality and persistent health inequality in middle-income countries: a case study of the Philippines. PLoS ONE.

[3] Do M, Soelaeman R, Hotchkiss DR (2015). Explaining inequity in the use of institutional delivery services in selected countries. Matern Child Health J.

[4] Ahmed S, Hill K (2011). Maternal mortality estimation at the subnational level: a model-based method with an application to Bangladesh. Bull World Health Organ.

[5] Mulholland EK, Smith L, Carneiro I (2008). Equity and child-survival strategies. Bull World Health Organ.

[6] Östlin P, Schrecker T, Sadana R (2011). Priorities for research on equity and health: towards an equity-focused health research agenda. PLoS Med.

[7] Bellows NM, Bellows BW, Warren C (2011). Systematic review: the use of vouchers for reproductive health services in developing countries: systematic review. Trop Med Int Health.

[8] Huntington D, Banzon E, Recidoro ZD (2012). A systems approach to improving maternal health in the Philippines. Bull World Health Organ.

[9] Philippine Statistics Authority [Philippines], ICF International (2014). National demographic and health survey, 2013.

[10] Oaxaca R (1973). Male–female wage differentials in urban labor markets. Int Econ Rev.

[11] Blinder AS (1973). Wage discrimination: reduced form and structural estimates. J Hum Resour.

[12] Hsiou TR, Pylypchuk Y (2012). Comparing and decomposing differences in preventive and hospital care: USA versus Taiwan. Health Econ.

[13] Lhila A, Long S (2012). What is driving the black-white difference in Low birthweight in the US?. Health Econ.

[14] Bhalotra S, Valente C, van Soest A (2010). The puzzle of Muslim advantage in child survival in India. J Health Econ.

[15] Finks JF, Osborne NH, Birkmeyer JD (2011). Trends in hospital volume and operative mortality for high-risk surgery. N Engl J Med.

[16] Fairlie RW (1999). The absence of the African-American owned business: an analysis of the dynamics of self-employment. J Labor Econ.

[17] Fairlie RW (2005). An extension of the blinder-Oaxaca decomposition technique to logit and probit models. J Econ Soc Meas.

[18] Darmstadt GL, Bhutta ZA, Cousens S, et al. Evidence-based, cost-effective interventions: how many newborn babies can we save? Lancet. 2005; 365(9463):977–88. doi:0.1016/S0140-6736(05)71088-6 10.1016/S0140-6736(05)71088-6 15767001

[19] Lawn JE, Cousens S, Zupan J (2005). Neonatal survival 1–4 million neonatal deaths: When? Where? Why?. Lancet.

[20] Thomas D, Messerschmidt L, Mersserschmidt D (2004). Increasing access to essential obstetric care: a review of progress and process.

[21] Thapa N, Chongsuvivatwong V, Geater AF (2000). High-risk childbirth practices in remote Nepal and their determinants. Women Health.

[22] Jimenez-Soto E, La Vincente S, Clark A, et al. Investment case for improving maternal and child health: results from four countries. BMC Public Health. 2013;13:601. doi:10.1186/1471-2458-13-601. Accessed 29 Sept 2015. 10.1186/1471-2458-13-601 PMC3701475 23800035

[23] New ERA (2007). Barriers and enabling factors influencing the use of a skilled birth attendant among marginalized populations in the Mid-Western region of Nepal.

[24] Thapa S (1996). Challenges to improving maternal health in rural Nepal. Lancet.

[25] Manandhar M (2000). Ethnographic perspectives on obstetric health issues in Nepal: a literature review.

[26] Filmer D, Pritchett LH (2001). Estimating wealth effects without expenditure data - or tears: an application to educational enrollments in states of India. Demography.

[27] Philippine Statistics Authority. Poverty statistics. Manila, The Philippines: Philippine Statistics Authority; 2015. http://psa.gov.ph/poverty-press-releases. Accessed 1 Jun 2016.

[28] Gabrysch S, Campbell OMR (2009). Still too far to walk: literature review of the determinants of delivery service use. BMC Pregnancy Childbirth.

[29] Agha S, Carton TW (2011). Determinants of institutional delivery in rural Jhang, Pakistan. Int J Equity Health.

[30] Kesterton AJ, Cleland J, Sloggett A (2010). Institutional delivery in rural India: the relative importance of accessibility and economic status. BMC Pregnancy Childbirth.

[31] Ndao-Brumblay SK, Mbaruku G, Kruk ME. Parity and institutional delivery in rural Tanzania: a multilevel analysis and policy implications. Health Policy and Planning. 2012. doi:10.1093/heapol/czs104 10.1093/heapol/czs104 PMC3753883 23132915

[32] Oaxaca RL, Ransom MR (1999). Identification in detailed wage decompositions. Rev Econ Stat.

[33] Furuta M, Salway S (2006). Women’s position within the household as a determinant of maternal health care use in Nepal. Int Fam Plan Perspect.

[34] Addai I (2000). Determinants of use of maternal-child health services in rural Ghana. J Biosoc Sci.

[35] Gage AJ, Guirlene CM (2006). Effects of the physical accessibility of maternal health services on their use in rural Haiti. Popul Stud (Camb).

[36] Navaneetham K, Dharmalingam A (2002). Utilization of maternal health care services in Southern India. Soc Sci Med.

[37] Stephenson R, Tsui AO (2002). Contextual influences on reproductive health service use in Uttar Pradesh, India. Stud Fam Plann.

[38] Philippine Health Insurance Corporation. PhilHealth Circular Number 15: The New PhilHealth Maternity Care Package For Normal Spontaneous Delivery (NSD) Performed in Accredited Hospitals. 2003. Pasig City, The Philippines: http://www.philhealth.gov.ph/circulars/2003/circ15_2003.pdf

[39] Philippine Health Insurance Corporation. PhilHealth Circular Number 23: Expanded Coverage of PhilHealth Normal Spontaneous Delivery Package in PhilHealth Accredited Hospitals and Maternity Care Package in PhilHealth Accredited Non-Hospital Facilities (Lying-In Clinics). 2006. Pasig City, The Philippines: http://www.philhealth.gov.ph/circulars/2006/circ23_2006.pdfAccessed 10 Aug 2015.

[40] Philippine Health Insurance Corporation. PhilHealth Circular Number 20: Expanded Coverage of PhilHealth Normal Spontaneous Delivery/Maternity Care Package In PhilHealth Accredited Hospitals and Non-hospital Facilities (Lying-In Clinics). 2008. Pasig City, The Philippines: http://www.philhealth.gov.ph/circulars/2008/circ20_2008.pdfAccessed 10 Aug 2015.

[41] Philippine Health Insurance Corporation. PhilHealth Circular Number 22: Social Health Insurance Coverage and Benefits for Women About to Give Birth. 2014. Pasig City, The Philippines: http://www.philhealth.gov.ph/circulars/2014/circ22_2014.pdfAccessed 10 Aug 2015.

[42] Thaddeus S, Maine D (1994). Too far to walk: maternal mortality in context. Soc Sci Med.

[43] Mwaliko E, Downing R, O’Meara W (2014). “Not too far to walk”: the influence of distance on place of delivery in a western Kenya health demographic surveillance system. BMC Health Serv Res.

[44] Hodge A, Byrne A, Morgan A (2015). Utilisation of health services and geography: deconstructing regional differences in barriers to facility-based delivery in Nepal. Matern Child Health J.

[45] Chi PC, Bulage P, Urdal H (2015). A qualitative study exploring the determinants of maternal health service uptake in post-conflict Burundi and Northern Uganda. BMC Pregnancy Childbirth.

[46] Price JI, Bohara AK. Maternal health care amid political unrest: the effect of armed conflict on antenatal care utilization in Nepal. Health Policy and Planning. 2012. doi:10.1093/heapol/czs062 10.1093/heapol/czs062 22773608

[47] Glei DA, Goldman N, Rodriguez G (2003). Utilization of care during pregnancy in rural Guatemala: does obstetrical need matter?. Soc Sci Med.

[48] Gyimah SO, Takyi BK, Addai I (2006). Challenges to the reproductive-health needs of African women: on religion and maternal health utilization in Ghana. Soc Sci Med.

[49] Government of the Philippines (2014). State of the nation address 2014 technical report, Philhealth enrolment.

[50] Government of the Philippines (2013). The responsible parent and reproductive health Act 2012, regulations of the republic Act 10354.

